# Inhibition of Dual/Mixed Tropic HIV-1 Isolates by CCR5-Inhibitors in Primary Lymphocytes and Macrophages

**DOI:** 10.1371/journal.pone.0068076

**Published:** 2013-07-09

**Authors:** Matteo Surdo, Emanuela Balestra, Patrizia Saccomandi, Fabiola Di Santo, Marco Montano, Domenico Di Carlo, Loredana Sarmati, Stefano Aquaro, Massimo Andreoni, Valentina Svicher, Carlo Federico Perno, Francesca Ceccherini-Silberstein

**Affiliations:** 1 Department of Experimental Medicine and Surgery, University of Rome Tor Vergata, Rome, Italy; 2 Clinical Infectious Diseases, Tor Vergata University (PTV), Rome, Italy; 3 Department of Pharmaco-Biology, University of Calabria, Rende (CS), Italy, 4INMI “L. Spallanzani”, Rome, Italy; Helmholtz Zentrum Muenchen - German Research Center for Environmental Health, Germany

## Abstract

**Background:**

Dual/mixed-tropic HIV-1 strains are predominant in a significant proportion of patients, though little information is available regarding their replication-capacity and susceptibility against CCR5-antagonists *in-vitro*. The aim of the study was to analyze the replication-capacity and susceptibility to maraviroc of HIV-1 clinical isolates with different tropism characteristics in primary monocyte-derived-macrophages (MDM), peripheral-blood-mononuclear-cells (PBMC), and CD4^+^T-lymphocytes.

**Methods:**

Twenty-three HIV-1 isolates were phenotipically and genotipically characterized as R5, X4 or dual (discriminated as R5^+^/X4, R5/X4, R5/X4^+^). Phenotypic-tropism was evaluated by multiple-cycles-assay on U87MG-CD4^+^-CCR5^+^−/CXCR4^+^-expressing cells. Genotypic-tropism prediction was obtained using Geno2Pheno-algorithm (false-positive-rate [FPR] = 10%). Replication-capacity and susceptibility to maraviroc were investigated in human-primary MDM, PBMC and CD4^+^T-cells. AMD3100 was used as CXCR4-inhibitor. Infectivity of R5/Dual/X4-viruses in presence/absence of maraviroc was assessed also by total HIV-DNA, quantified by real-time polymerase-chain-reaction.

**Results:**

Among 23 HIV-1 clinical isolates, phenotypic-tropism-assay distinguished 4, 17 and 2 viruses with R5-tropic, dual/mixed-, and X4-tropic characteristics, respectively. Overall, viruses defined as R5^+^/X4-tropic were found with the highest prevalence (10/23, 43.5%). The majority of isolates efficiently replicated in both PBMC and CD4^+^T-cells, regardless of their tropism, while MDM mainly sustained replication of R5- or R5^+^/X4-tropic isolates; strong correlation between viral-replication and genotypic-FPR-values was observed in MDM (rho = 0.710;p-value = 1.4e-4). In all primary cells, maraviroc inhibited viral-replication of isolates not only with pure R5- but also with dual/mixed tropism (mainly R5^+^/X4 and, to a lesser extent R5/X4 and R5/X4^+^). Finally, no main differences by comparing the total HIV-DNA with the p24-production in presence/absence of maraviroc were found.

**Conclusions:**

Maraviroc is effective *in-vitro* against viruses with dual-characteristics in both MDM and lymphocytes, despite the potential X4-mediated escape. This suggests that the concept of HIV-entry through one of the two coreceptors “separately” may require revision, and that the use of CCR5-antagonists in patients with dual/mixed-tropic viruses may be a therapeutic-option that deserves further investigations in different clinical settings.

## Introduction

The entry of human immunodeficiency virus (HIV) into human primary cells, such as lymphocytes and macrophages, is a complex multistep process that can be targeted for antiretroviral drugs. This process needs the interaction between viral proteins and cellular receptors: in particular the first important interaction occurs between the viral glycoprotein gp120 and the CD4 cellular receptor. In addition, other specific co-receptors, CCR5 and/or CXCR4, interact with the viral gp120 protein, inducing specific conformational changes that facilitate the viral entry into the cell mediated by the fusion protein gp41. These cellular chemokine receptors are G protein characterized by seven-transmembrane domains and are able to determine the cellular tropism of HIV-1. Pure R5 viruses utilize only the CCR5 co-receptor to enter into target cells, while pure X4 tropic viruses utilize only the CXCR4 co-receptor [1]–[6]. Some viruses are able to use both coreceptors to enter into the cell, and are defined dual-tropic [7]. Recent studies highlighted the existence of different types of dual-tropic viruses: those that are more efficient in using the CCR5 co-receptor (R5^+^/X4), those that use more efficiently the CXCR4 co-receptor (R5/X4^+^), and those that can use with the same efficiency both co-receptors (R5/X4) [8]. For historical reasons, R5 viruses are often classified also as macrophage M- tropic viruses because of their propensity to infect these cells. Conversely, X4 strains are named lymphocytic T-tropic, because on their pronounced replication capacity on such type of cells [9], [10].

The tropism is based upon the presence of selected amino acids in gp120 (particularly within the V3 loop, but not only) and gp41 glycoproteins, that provide greater affinity to use CCR5 or CXCR4 [11]. In the majority of patients, R5 variants predominate during the early stage of infection, but the progression of the disease is associated to an increase of the CXCR4 using virus [5], [12]–[18]. This natural shift in viruses using also the CXCR4 coreceptor is fundamental in the emergence of dual-tropic viruses in patient’s viral population, that represents approximately the 15%–25% of naïve patients and the 25%–40% of experienced patients carrying HIV-1 B subtype [12], [19]–[22]. Conversely, the prevalence of pure X4 tropic viruses is 0.1% in naïve patients and 2–3% in treated patients, always carrying HIV-1 B subtype [12], [21], [23]. The viral coreceptor usage can be phenotypically determined by the approved tropism test, the Trofile assay (Monogram Biosciences) and its newer iteration, the Enhanced Sensitivity Trofile Assay (ESTA). This test is based on a single-cycle recombinant virus assay that uses pseudovirus with full-length *env-*genes from the patient’s virus population to infect target cell lines engineered to express CD4 and either the CCR5 or CXCR4 chemokine co-receptor. The genotypic test, also now available for tropism determination in clinical practice, is a rapid method based on the sequence of the third variable (V3) loop of the HIV gp120. The sequence is analyzed by using bio-informatic algorithms, such as PSSM_X4/R5_ and geno2pheno_[coreceptor]_ (G2P) [24]–[27]. Furthermore, with the advent of next-generation deep sequencing, with a much higher sensitivity, today it is possible to detect minority variants of R5 and non-R5 viruses (starting from 0.1% of prevalence) also with a genotypic test [28]–[30].

Maraviroc is a recently Food and Drug Administration (FDA) approved antiretroviral drug with a novel mechanism of action [31], [32]. It can be used for the treatment of HIV drug-naïve and drug-experienced patients, and also very important, of patients with multi-resistant viruses to other antiretroviral drugs [32]–[35]. It acts by binding to the CCR5 coreceptor within a cavity located among the membrane-spanning helices, and it stabilizes the receptor in a conformation not efficiently recognized by the viral gp120, thus preventing HIV-1 entry [36]. Based on this specific mechanism of action, maraviroc is recommended only in patients infected by pure R5 viruses (http://aidsinfo.nih.gov/contentfiles/lvguidelines/adultandadolescentgl.pdf).

So far, very few studies have focused on the *in vitro* and *in vivo* efficacy of maraviroc against HIV-1 dual-tropic viruses [28], [37]–[39]. Similarly, very few studies also analyzed the replication capacity of HIV-1 dual-tropic viruses in human primary cells, such as CD4+ T-cells and macrophages, that are the two principal targets and sanctuaries of HIV infection [40]–[43]. Indeed, macrophages can sustain viral infection for long periods of time, from weeks to months, *in vitro* and *in vivo* [44]–[49] and they can efficiently transfer virus to CD4+ T-lymphocytes contributing to their depletion in human cellular compartments [50].

It was recently recognized that R5 HIV-1 isolates can markedly vary in their replication capacity in macrophages and that some X4 viruses are also capable of replication in these cells [51].

For all of these reasons, the innovative aim of the study was to analyze both the replication capacity and the *in vitro* efficacy of maraviroc against clinical isolates with different tropic characteristics in human primary macrophages, peripheral blood mononuclear cells (PBMC) and lymphocytes. In particular, we tested the activity of entry antagonists against several dual-tropic viruses with a wide range of phenotypic and genotypic tropic preferences.

## Materials and Methods

### Cells

Human astroglioma U87MG-cells expressing CD4-receptor alone or with CXCR4 co-receptor (U87MG-CD4^+^/CXCR4^+^) or CCR5 co-receptor (U87MG-CD4^+^/CCR5^+^) were kindly obtained through the AIDS Research and Reference Reagent Program, Division of AIDS, NIAID, NIH: from Dr. Hong Kui Deng and Dr. Dan R. Littman [52]. U87MG-parental cells were obtained through the AIDS Research and Reference Reagent Program, Division of AIDS, NIAID, NIH: from Dr. Bruce Chesebro [53]. All these cells were maintained in DMEM (Euroclone) with the addition of 15% heat-inactivated, mycoplasma- and endotoxin- free fetal bovine serum (FBS) (HyClone), 1 mM sodium pyruvate (Euroclone), 0.1 mM non-essential amino acids (Euroclone), 100 U/ml penicillin +100 µg/ml streptomycin (Euroclone) and 2 mM L-glutamine (Euroclone). Medium for U87MG-CD4^+^, U87MG-CD4^+^/CXCR4^+^ and U87MG-CD4^+^/CCR5^+^ was supplemented with 300 µg/ml G418 (Sigma-Chemicals). Only for the CCR5 and CXCR4-*expressing* cells the medium was supplemented also with 1ug/ml of puromycin (Sigma-Aldrich).

PBMC and Monocyte-derived Macrophages (MDM) were obtained from the blood of healthy seronegative donors by separation over Ficoll-Hypaque gradient, as previously described [54], [55]. Ethic approval was deemed unnecessary because, under Italian law, biomedical research is subjected to previous approval by ethics committees only in the hypothesis of clinical trials on medicinal products for clinical use (art. 6 and art. 9, leg. decree 211/2003). Also, the research was conducted on blood samples previously anonymized, according to the requirements set by Italian Data Protection Code (leg. decree 196/2003). The research was not conducted outside of the country of residence. PBMC were seeded in T25 flask (Costar) at a density of 1.0×10^6^ cells/ml in RPMI 1640 (Euroclone) supplemented with 100 U/ml penicillin +100 µg/ml streptomycin, 2 mM L-glutamine, and 10% FBS in addition to 2 µg/ml of phytohemagglutinin (PHA, Sigma) and 50 U/ml recombinant interleukin-2 (IL-2, Peprotech).

In order to prepare MDM, PBMC were resuspended in RPMI 1640 medium supplemented with 20% FBS then seeded into 48-well plates (1.8×10^6^ cells/well). After 5 days, non-adherent cells were carefully removed by repeated washings. MDMs were estimated to be 10^5^/well at the time of infection. Adherent cells obtained using this technique generally consisted of pure differentiated MDM (at cytofluorimetric analysis, more than 95% of cells were CD14^+^/CD4^+^/CD3^−^).

Primary CD4+ T-cells were highly enriched via negative selection of PBMC using the Human CD4^+^ T Cell enrichment kit (StemCell EasySep)**.** The isolated CD4^+^ T cells were seeded in T25 flask at a density of 2.0×10^6^ cells/ml in RPMI 1640 medium supplemented with 20% FBS, penicillin (100 U/ml), streptomycin (100 µg/ml) and L-glutamine (2 mM). The cells were activated and stimulated with 50 U/ml rIL-2 and 5 µg/ml PHA for 2–3 days before infection. The purity of CD4+ T-cells was evaluated through cytofluorimetric analysis (generally more than 95–98% of cells were CD4^+^/CD8−/CD14^−^/CCR5^+^/CXCR4^+^).

### Compounds

The CCR5 inhibitor maraviroc (Selzentry, MVC) and Nucleoside Reverse Transcriptase Inhibitor (NRTI) 3′-azido-2′, 3′-dideoxythymidine (AZT, used as a control drug) were obtained from NIH AIDS Research and Reference Reagent Program. The CXCR4 inhibitor AMD3100 was synthesized by SIGMA.

### Viruses

Twenty-three isolates were obtained expanding primary isolates from CD8^+^-depleted PBMC of naïve- or HAART-experienced HIV-infected patients, as previously described [56], [57]. All isolates used in the study were previously characterized for the tropism characteristics [8]. The laboratory strains HIV_BaL_ (pure R5-tropic) and HIV_IIIB_ (pure X4-tropic) were used as controls in all *in-vitro* experiments.

### Phenotypic-tropism

Viral tropism was evaluated by a phenotypic assay based on multiple cycles of replication in astroglioma U87MG-CD4^+^/CCR5^+^ and U87MG-CD4^+^/CXCR4^+^ cells [8]. Briefly, U87MG-CD4^+^/CCR5^+^ and U87MG-CD4^+^/CXCR4^+^ cells (2×10^5^ cells/ml) were grown in 96-well plates in a final volume of 200 µl/well. After 24 hours, the medium was removed, and the infection was performed by using 100 µl/well of viral suspension (1,200 p24 pg/w) for 2 hours. The test was performed in quadruplicate. After 14 days of culture, the supernatants were collected and analyzed for p24 production by a commercial ELISA kit, according to the manufacturer (Ag-Genscreen TM HIV-1 Assay, BioRad).

### *In-vitro* Efficacy of Maraviroc

The antiviral activity of maraviroc was evaluated using three different concentrations: 200 nM, 20 nM and 2 nM, corresponding to 100X, 10X and 1X the IC_50_ [31], respectively. Zidovudine (AZT) was used as a control at a concentration of 1000 nM, known to inhibit >99% the replication of laboratory strains [58], AMD3100 was used at a concentration of 1300 nM, known to inhibit >99% the replication of laboratory X4-*using* strain HIV_IIIB_ [59]. Stimulated PBMC and CD4+ T-cells were infected with a standardized input of each virus (16,000 p24 pg/ml/10^6^cells). After 2 hours of adsorption, cells were extensively washed to remove any residual viral particle, re-suspended in complete medium (1 ml) and cultured on 48-well plates (500,000 cells/well) for 7 days. All the drugs were always present in treated wells in 1 hour-pretreatment phase, during infection stage and for the 7 days later. Virus-growth was monitored at the day 7 post infection by measuring supernatant p24 levels, as described above. All assays were performed at least in duplicate wells. Each isolate was tested at least in 3 independent experiments and infected-PBMC were also kept untreated, as a control of viral replication without drug-pressure. Primary MDM were infected with a standardized input of each virus (8,000 p24 pg/ml/10^5^cells). After 24 hours of adsorption, cells were extensively washed to remove any residual viral particle, then new complete medium (1 ml) was added and cells were cultured for 7 days. At day 7 post infection cells were washed again and cultured for another 7 days with 1 ml of new complete medium. All the drugs were always present in treated wells in 1 hour-pretreatment phase, during infection stage and for the 14 days later. Virus-growth was monitored at the day 14 post infection by measuring supernatant p24 levels by ELISA kit. All assays were performed in triplicate. Each isolate was tested at least in 3 independent experiments and, infected-MDM were also kept untreated to control for viral replication without drug-pressure.

### Genotypic Analysis

The V3 analysis was performed according to previous publication [8]. Briefly, HIV-1 RNA was extracted from each isolate with a commercially available kit (QIAamp RNA Viral Mini kit, Qiagen). The HIV-1 V3 sequence was amplified by two overlapping polymerase chain reactions (PCR) using two primers S2∶5′CACAGTACAATGTACACA 3′ and AS5∶5′CTTCTCCAATTGTCCCTCA3′). Primers A = S2∶5′CAGCACAGTACAATGTACACA3′; B = S5∶5′GTTAAATGGCAGTCTAGCAG3′; C = AS1∶5′GAAAAATTCCCCTCCACAATT3′ and D = AS3bis:5′CAATTTCTGGGTCCCCTC3′ were used to sequence PCR-products in both directions on an automated sequencer (ABI3100, Applied Biosystems). Translated protein sequences from gp120-genes were generated and aligned using Bioedit 7.0 software and CLUSTALW1.8. Nucleotidic V3 sequences of isolates are available as Table S1. Each V3 sequence was used to infer viral tropism using geno2pheno algorithm (available at http://coreceptor.bioinf.mpi-inf.mpg.de/; set at a false positive rate [FPR] of 10%) and using PSSM (available at http://fortinbras.us/cgi-bin/fssm/fssm.pl#avg; set at a cutoff of -5.96).

### Cell-Associated HIV-1 DNA Quantification

PBMC and MDM, infected and treated as mentioned above, were harvested, after gently washing with warm PBS, counted, dry pelleted and stored at −20°C. Total cell DNA was then extracted from cellular pellets with High Pure PCR Template Preparation Kit (Roche Molecular Biochemicals, Germany) and stored at −20°C. HIV-1 total DNA was quantified by real-time polymerase chain reaction, using 5′ nuclease assay in the long terminal repeat (LTR) region of HIV-1 DNA genome (reference sequence HXB2). The reaction was performed with a LightCycler v3.5 (Roche Molecular Biochemicals, Indianapolis, IN). Briefly, up to 500 ng of DNA was amplified with the sense primer NEC 152 (GCCTCAATAAAGCTTGCCTTGA) and the reverse primer NEC 131 (GGCGCCACTGCTAGAGATTTT) producing a 121 bp fragment, in the presence of a dually (FAM and TAMRA) labeled NEC LTR probe (AAGTAGTGTGTGCCCGTCTGTTRTKTGACT) published by Jean- Paul Viard et al [60]. The first PCR cycle allowing fluorescence detection permitted to quantify HIV-1 DNA by reference to a standard curve signal (sevenfold dilutions from 75,000 to 1.87 copies of 8e5 cell DNA containing one integrated HIV-1 DNA copy per cell [60], [61]) read on channel F1/F2 of the LightCycler. To verify DNA integrity, we used LC control DNA Kit reagents (Roche Molecular Biochemicals) to amplify a 110-bp fragment of human β-globine in the same reaction. This second internal control target was hybridised with a FRET probe and read on channel F3/F2 of the LightCycler. To verify the accuracy of the Real-Time PCR result, different HIV-1 DNA standards (AIDS Research and Reference Reagent Program, DAIDS, NIAID, NIH:PCR Panel 001 from Dr. Shirley Kwok and Dr. Cindy Christopherson, Roche Molecular Systems) were also quantified [60], [62]. Results were expressed as number of total HIV-1 DNA copies/10^6^ cells.

### Statistical Analysis

Comparisons of independent groups were made by Mann-Whitney-Wilcoxon (MWW) test. To correlate the replication capacity (p24 production) and genotypic tropism (values of Geno2Pheno FPR) as well as the p24 production and HIV-DNA content, simple linear regression model and Pearson correlation test have been performed by using the statistical environment R (version 2.15.1; [63]). P-values less than 0.05 were considered statistically significant.

## Results

### Genotypic and Phenotypic Characterization of Isolates

Twenty-three HIV-1 isolates were phenotypically and genotypically characterized. By the phenotypic-tropism evaluation on U87MG-CD4^+^-CCR5^+^ and U87MG-CD4^+^-CXCR4^+^-expressing cells, the isolates were divided into 4 pure R5-tropic viruses (17.4%), 17 dual/mixed-tropic viruses (73.9%), and only 2 X4-tropic viruses (8.7%). Among the 17 dual/mixed-isolates, 10 isolates were subsequently classified as R5^+^/X4, 5 isolates in R5/X4^+^, while 2 isolates showed a dual/mixed tropism (R5/X4) without any preference versus the chemokine coreceptors. The V3 sequence was successfully obtained for all 23 isolates. Interestingly, all 14 isolates with an R5 genotypic tropism prediction (geno2pheno FPR >10%) were phenotypically characterized either as pure R5 or R5^+^/X4 viruses, while the remaining isolates with a X4 genotypic prediction (FPR<10%) were all phenotypically defined as R5/X4, R5/X4^+^ or pure X4 tropic (Table 1), thus confirming a substantial homogeneity of genotypic versus phenotypic characterization. In addition, PSSM algorithm also confirmed these results showing a concordance of 91.3% with geno2pheno algorithm (only 2 discordances in the dual/mixed viruses were observed) (data not shown).

**Table 1:** Isolate’s genotypic and phenotypic characteristics.

Isolate ID	Genotypic tropism^a^		Phenotypic tropism
	FPR (%)	Prediction	Prediction^b^
**18**	95.2	R5	R5^+^/X4
**15**	94.6	R5	R5^+^/X4
**12**	75.6	R5	R5^+^/X4
**31**	58.7	R5	R5
**28**	53.5	R5	R5
**5**	52.8	R5	R5
**7**	50.5	R5	R5^+^/X4
**8**	49.2	R5	R5^+^/X4
**38**	48.0	R5	R5^+^/X4
**23**	45.7	R5	R5
**29**	34.3	R5	R5^+^/X4
**20**	28.8	R5	R5^+^/X4
**14**	16.9	R5	R5^+^/X4
**19**	12.1	R5	R5^+^/X4
**32**	4.7	X4	R5/X4
**4**	2.7	X4	R5/X4^+^
**9**	1.8	X4	R5/X4^+^
**1**	1.1	X4	R5/X4^+^
**34**	0.7	X4	R5/X4
**26**	0.4	X4	R5/X4^+^
**39**	0.2	X4	R5/X4^+^
**16**	0.2	X4	X4
**17**	0.1	X4	X4

a The V3 sequence was used to infer viral tropism using geno2pheno (set at a false positive rate [FPR] of 10%) algorithms.

b Phenotypic-tropism was evaluated by multiple-cycles-assay on astroglioma U87MG-CD4^+^-CCR5^+^/−CXCR4^+^-expressing cells.

### Viral Replication and Maraviroc Activity in PBMC

**Figure 1:**
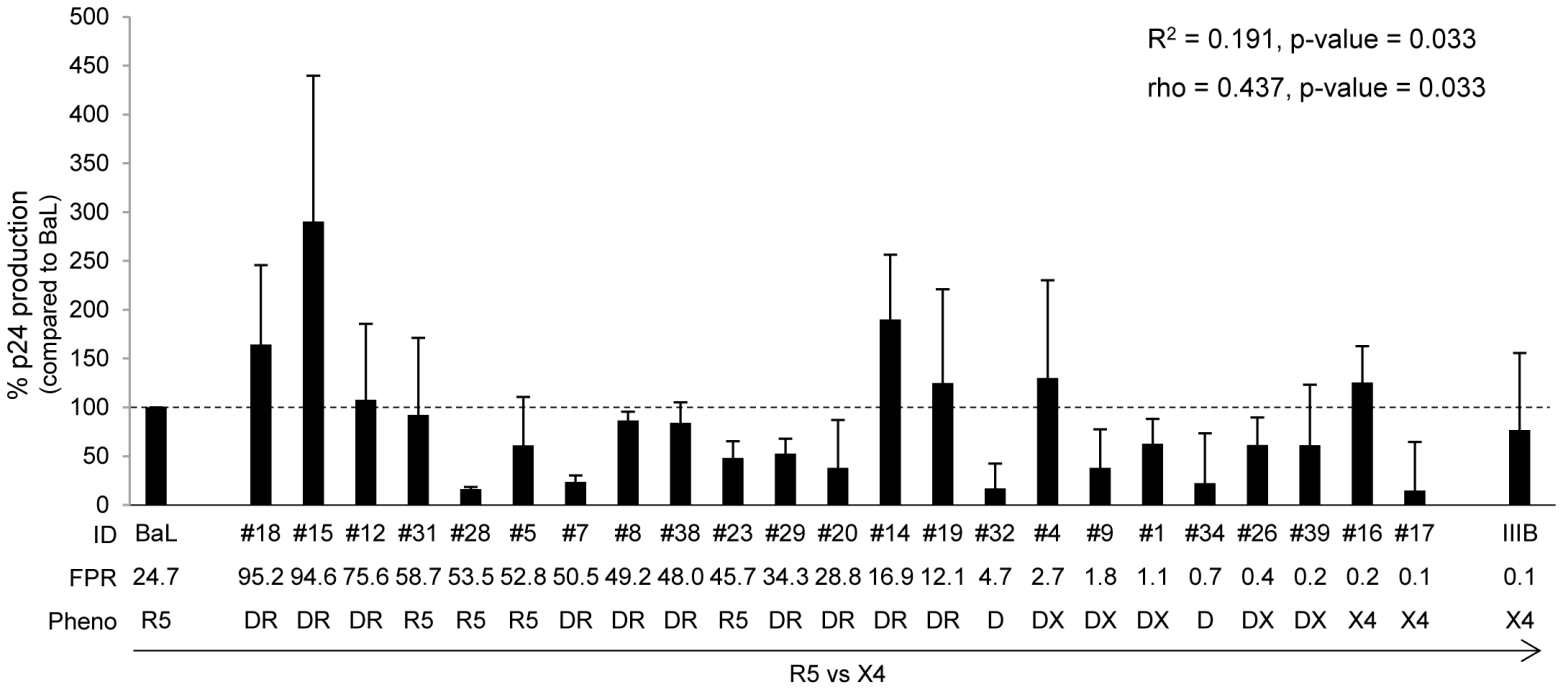
Replication capacity in PBMC. The bars represents the percentages of HIV-1 isolate replication in PBMC cultures infected with 16,000 p24 pg per 10^6^ cells after 7 days post infection. The percentage of viral replication of each isolate is calculated considering the replication of the control virus HIV_BaL_ as 100% (median value of 76,688 pg/ml). Mean values of at least 3 experiments for each isolate are shown. Error bars indicating inter-experimental standard deviations are shown. Linear regression model and Pearson correlation coefficient were used to determine the strength of correlation between the replication capacity (p24 production) and genotypic tropism (values of Geno2Pheno FPR). R^2^ = Coefficient of determination; rho = Pearson correlation coefficient; p, two-tailed p value. The False Positive Rate (FPR) values are based on the V3 sequences and calculated with Geno2Pheno algorithm. The phenotypic tropism was evaluated on astroglioma U87MG-CD4^+^-CCR5^+^−/CXCR4^+^-expressing cells (DR: R5^+^X4; D: R5/X4; DX: R5/X4^+^).

A goal of the study was to evaluate in different human primary cells the replication capacity of these isolates, according to their tropism preferences in presence and absence of entry-inhibitors. As control, the replication capacity of HIV laboratory strains, BaL (R5) and IIIB (X4), was also analyzed. In PBMC, both laboratory strains showed a similar p24 production at 7 days post infection, with a mean ± SD of 76,688±55,102 pg/ml versus 73,487±60,477 pg/ml, respectively. As shown in Figure 1, the majority of isolates, including dual/mixed, regardless of genotypic FPR values, efficiently replicated in PBMC (either in the range of, or more or lower than HIV-1_BaL_ control strain). Median HIV-1 gag-p24 antigen release in PBMC supernatants at 7 days post infection (compared to HIV-1_BaL_) was 61.4% (34,913 pg/ml; IQR: 18,848–71,949 pg/ml). Statistical analysis showed a direct correlation between viral replication and genotypic FPR values (R^2^ = 0.191, p-value = 0.033; rho = 0.437, p-value = 0.033).

Similar results were obtained with CD4+ T-cells, where median HIV-1 gag-p24 antigen release in their supernatants at 7 days post infection (compared to HIV-1_BaL_) was 62.9% (50,831 pg/ml; IQR: 10,146–92,302 pg/ml; 12 isolates tested) (data not shown). Only 3 isolates (#28, #32 and #17) poorly replicated (mean value <12.2% of p24 antigen release), and this phenomenon was independent of the viral tropism.

We then tested the replication capacity of HIV isolates in PBMC in presence of maraviroc (CCR5-antagonist), or AMD3100 (CXCR4-antagonist). The NRTI AZT has been used as control drug.

**Figure 2:**
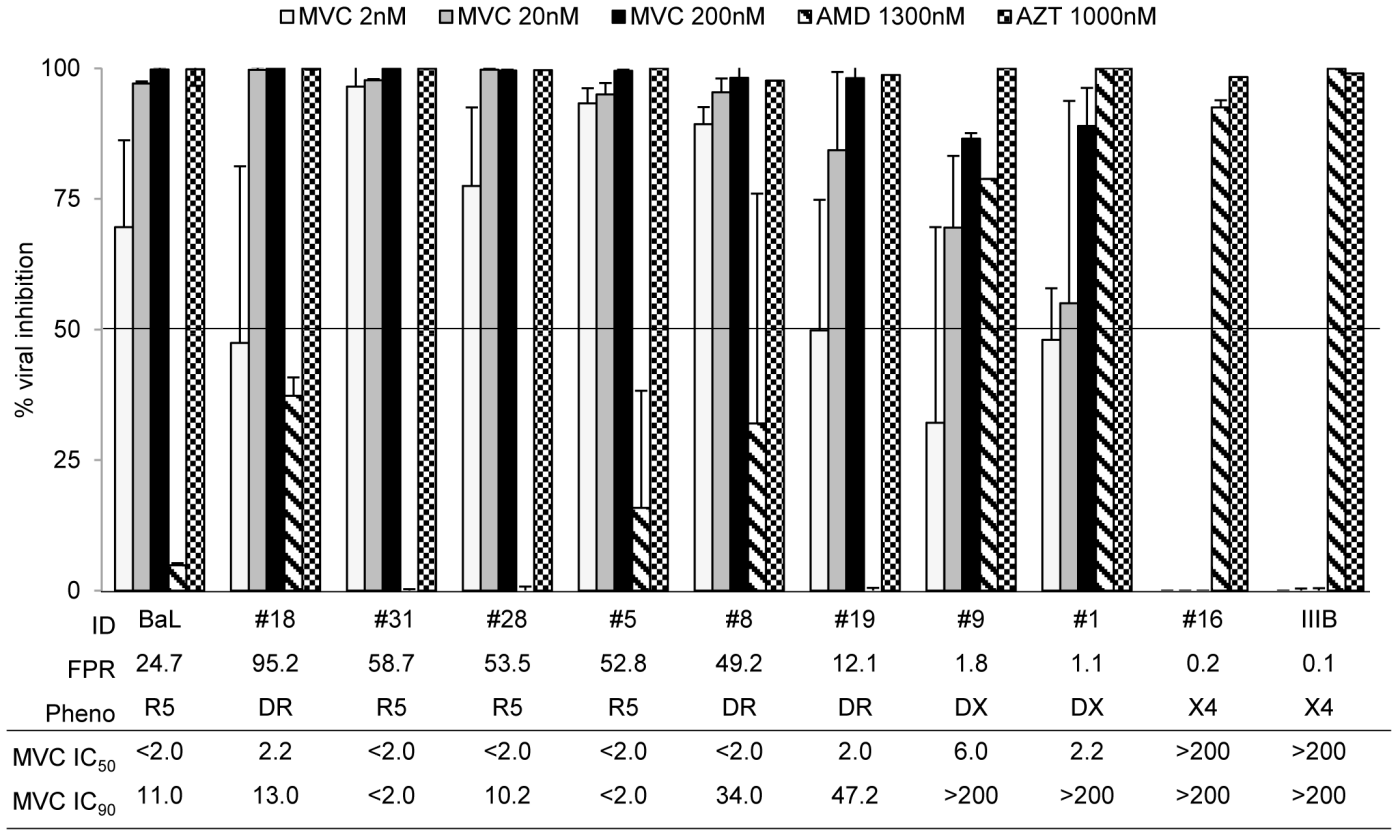
Activity of Maraviroc in PBMC. *In vitro* efficacy of Maraviroc in PBMC cultures. The histogram represents the percentage of viral inhibition (p24 production pg/ml) in presence of Maraviroc, AMD3100 and AZT at different concentrations, considering the untreated as 100. Mean values of at least 3 experiments for each isolate are shown. Error bars indicating inter-experimental standard deviations are shown. Maraviroc median IC_50_ and IC_90_ values of 3 different experiments are also shown. The False Positive Rate (FPR) values are based on the V3 sequences and calculated with Geno2Pheno algorithm. The phenotypic tropism was evaluated on astroglioma U87MG-CD4^+^-CCR5^+^−/CXCR4^+^-expressing cells (DR: R5^+^X4; D: R5/X4; DX: R5/X4^+^).

All isolates responded to at least one of the two chemokine coreceptor antagonists, with a dose response modulation. Maraviroc was highly effective in PBMC (Figure 2) against all tested isolates with R5-genotypic prediction (FPR>10%, n = 6, including 3 R5^+^/X4), showing a median [IQR] inhibition of the viral replication of 99.9% [98.5–99.8] at 200 nM, 97.0% [95.1–99.2] at 20 nM, and 83% [56.7–92.3] at 2 nM.

In all these isolates, the median maraviroc IC_50_ was ≤2 nM, and the median IC_90_ for each isolate ranged from <2 nM to 47.2 nM. Somewhat to our surprise, the 2 R5/X4^+^ isolates with FPR<5% (#1 and #9), tested in the study, were also inhibited by maraviroc, though less efficiently than the previous ones (median inhibition of 89.9% at 200 nM, 63.0% at 20 nM, 42.0% at 2 nM) (Figure 2). In these two isolates, maraviroc IC_50_ was 2.2 nM and 6 nM, respectively, and the IC_90_ was always >200 nM. As expected, maraviroc did not inhibit the replication of the pure-X4 isolate (#16). Conversely, AMD3100 at a concentration of 1300 nM was active against the pure-X4 isolate (#16), and also against the dual/mixed isolates, particularly efficient with those having FPR <5% (median [IQR] inhibition of 92.5% [85.7–96.3] (Figure 2). As expected, AMD3100 did not consistently inhibit the replication of 2 out of 3 pure-R5 isolates (inhibition activity <1%), while very poorly inhibited 1 R5-isolate (inhibition activity of 16%). As expected, AZT, used as control, inhibited all strains at the used concentration of 1000 nM.

Thus, the majority of isolates, regardless of their tropism, efficiently replicated in both PBMC and CD4+ T-cells and maraviroc was active in PBMC against also dual/mixed viruses despite the potential X4-mediated escape.

### Viral Replication and Maraviroc Activity in MDM

**Figure 3:**
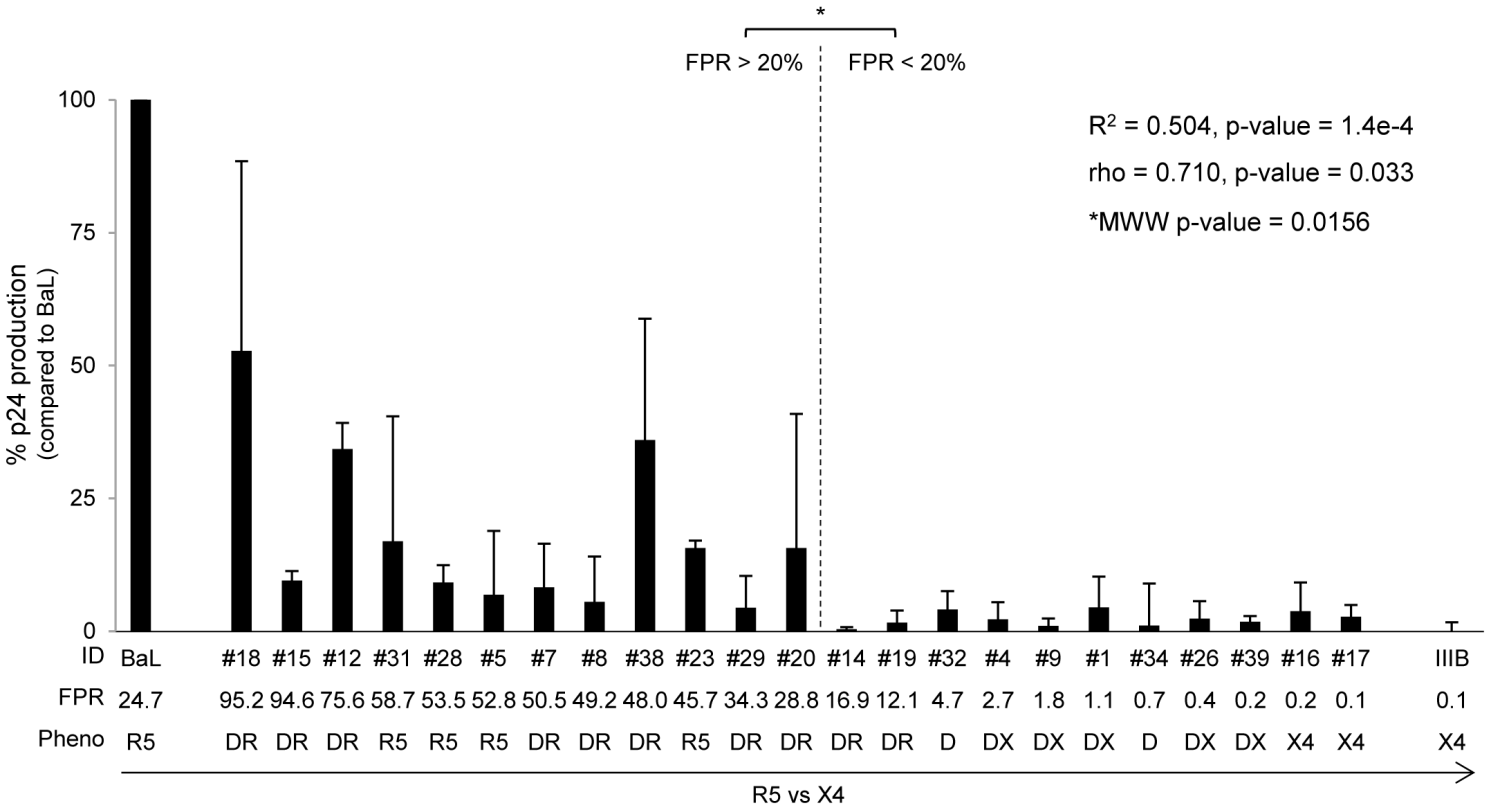
Replication capacity in MDM. The bars represents the percentages of HIV-1 isolate replication in MDM cultures infected with 8,000 pg per 10^5^ cells after 14 days post infection. The percentage of viral replication of each isolate is calculated considering the replication of the control virus HIV_BaL_ as 100% (median value of 162,143 pg/ml). Mean values of at least 3 experiments for each isolate are shown. Error bars indicating inter-experimental standard deviations are shown. Linear regression model and Pearson correlation coefficient were used to determine the strength of correlation between the replication capacity (p24 production) and genotypic tropism (values of Geno2Pheno FPR). Mann-Whitney-Wilcoxon (MWW) test was used to compare the replication capacity between isolates with FPR <20% and >20%. R^2^ = Coefficient of determination; rho = Pearson correlation coefficient; p, two-tailed p value. The False Positive Rate (FPR) values are based on the V3 sequences and calculated with Geno2Pheno algorithm. The phenotypic tropism was evaluated on astroglioma U87MG-CD4^+^-CCR5^+^−/CXCR4^+^-expressing cells (DR: R5^+^X4; D: R5/X4; DX: R5/X4^+^).

The replication capacity of isolates and control strains was also tested in MDM at 14 days post infection in presence and absence of antiviral compounds. As expected, in MDM, the replication capacity of HIV-1_BaL_ control strain was very efficient, producing a mean ± SD of 162,143±127,019 pg/ml, while replication of HIV-1_IIB_ was barely detected. By statistical analysis we observed a strong and significant direct correlation between viral replication and genotypic FPR values (R^2^ = 0.504, p-value = 1.4e-4; rho = 0.710, p-value = 1.4e-4) (Figure 3). In particular, isolates with FPR>20%, phenotypically defined as pure R5- or R5^+^/X4-viruses, replicated with higher efficiency compared to isolates with FPR<20%, (median p24 = 8,929 pg/ml, IQR: 3,948–14,124 pg/ml versus 3,310 pg/ml, IQR: 2,325–5,451 pg/ml, respectively; MWW, p-value = 0.0156), confirming that macrophages sustained mainly the replication of isolates with preferential CCR5-usage (Figure 3).

**Figure 4:**
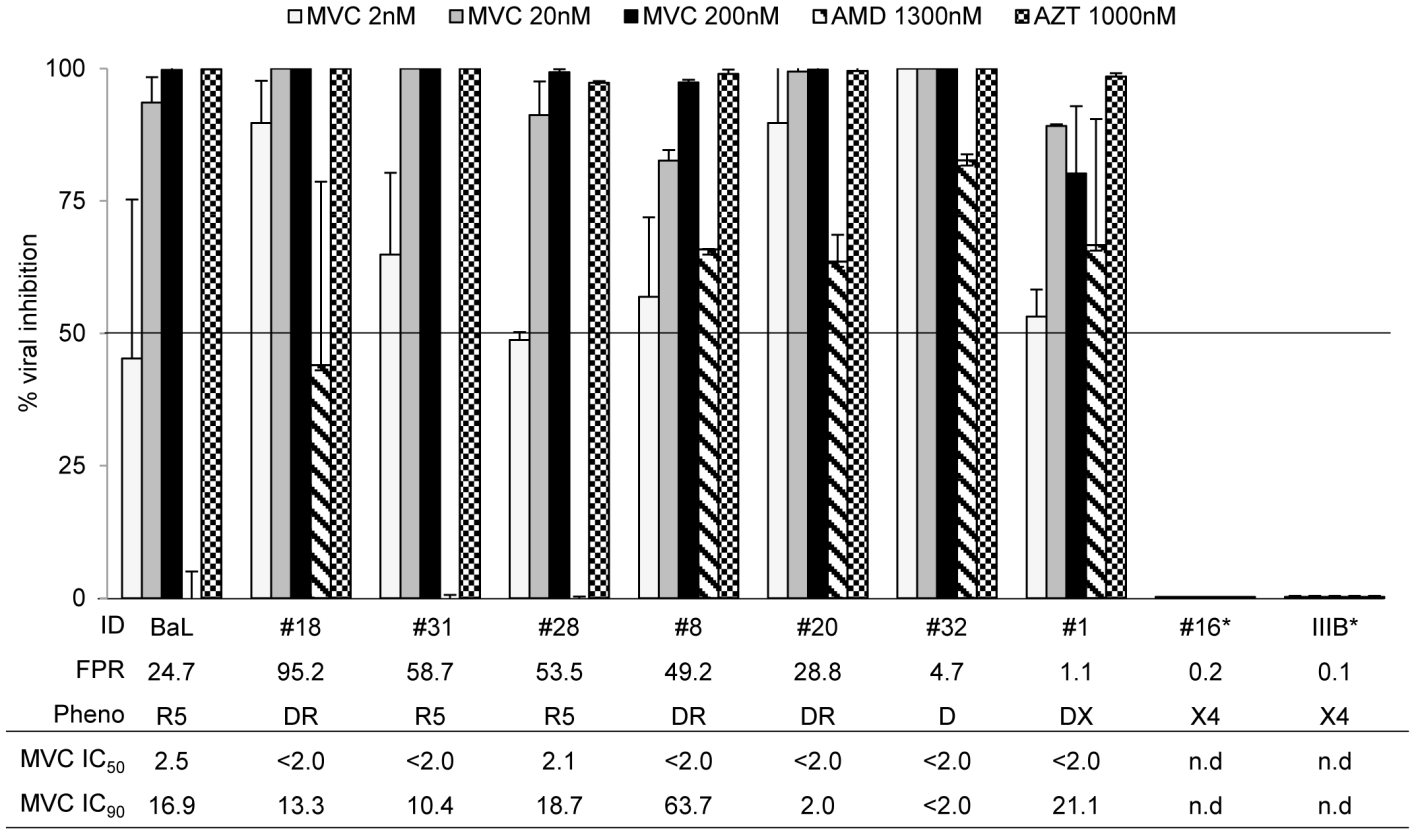
Activity of Maraviroc in MDM. *In vitro* efficacy of Maraviroc in MDM cultures. The bars represents the percentage of viral inhibition (p24 production pg/ml) in presence of Maraviroc, AMD3100 and AZT at different concentrations, considering the untreated as 100. Mean values of at least 3 experiments for each isolate are shown. Error bars indicating inter-experimental standard deviations are shown. Maraviroc median IC_50_ and IC_90_ values of 3 different experiments are also shown. The False Positive Rate (FPR) values are based on the V3 sequences and calculated with Geno2Pheno algorithm. The phenotypic tropism was evaluated on astroglioma U87MG-CD4^+^-CCR5^+^−/CXCR4^+^-expressing cells (DR: R5^+^X4; D: R5/X4; DX: R5/X4^+^).*Antiviral activity against X4-tropic viruses (#16 and HIV_IIIB_) was not evaluated because due to their low replication.

The activity of maraviroc, AMD3100 and the AZT was also analyzed in MDM (Figure 4). As observed with PBMC, maraviroc was highly effective against all tested isolates with FPR>10% (n = 5, including 3 R5^+^/X4), showing a median [IQR] inhibition of the viral replication of 99.5% [97.8–99.9] at 200 nM, 95.3% [84.8–99.8] at 20 nM, and 52.8% [45.2–62.8] at 2 nM (Figure 4). Of interest, also the only 2 dual-tropic isolates with FPR<5%, 1 R5/X4^+^ (#1) and 1 R5/X4 (#32), that both sufficiently replicated (median p24 of 3 experiments = 2,135 pg/ml and 4,567 pg/ml, respectively) and therefore tested in MDM, were inhibited by maraviroc (median [IQR] inhibition of 90.1% [85.2–95.1] at 200 nM, 94.6% [91.9–95.1] at 20 nM, 76.6% [64.9–97.3] at 2 nM). In all tested isolates, MDM’s maraviroc median IC_50_ was ≤2 nM, and the median IC_90_ ranged from 2 nM to 63.7 nM. By contrast, AMD3100 at a concentration of 1300 nM did not inhibit R5-viruses, but inhibited the replication of dual-tropic viruses at a median [IQR] of 79.4% [73.0–81.0], with a particular efficiency in the 2 dual-tropic isolates with FPR<5%, (Figure 4).

Taken together, these results show that macrophages mainly sustain the replication of HIV-1 isolates with pure R5 or R5^+^/X4 tropism, and that maraviroc is highly active against all tested isolates.

### Evaluation of HIV Total DNA Expression in Presence or Absence of Entry Inhibitors in Human Primary Cells

**Figure 5:**
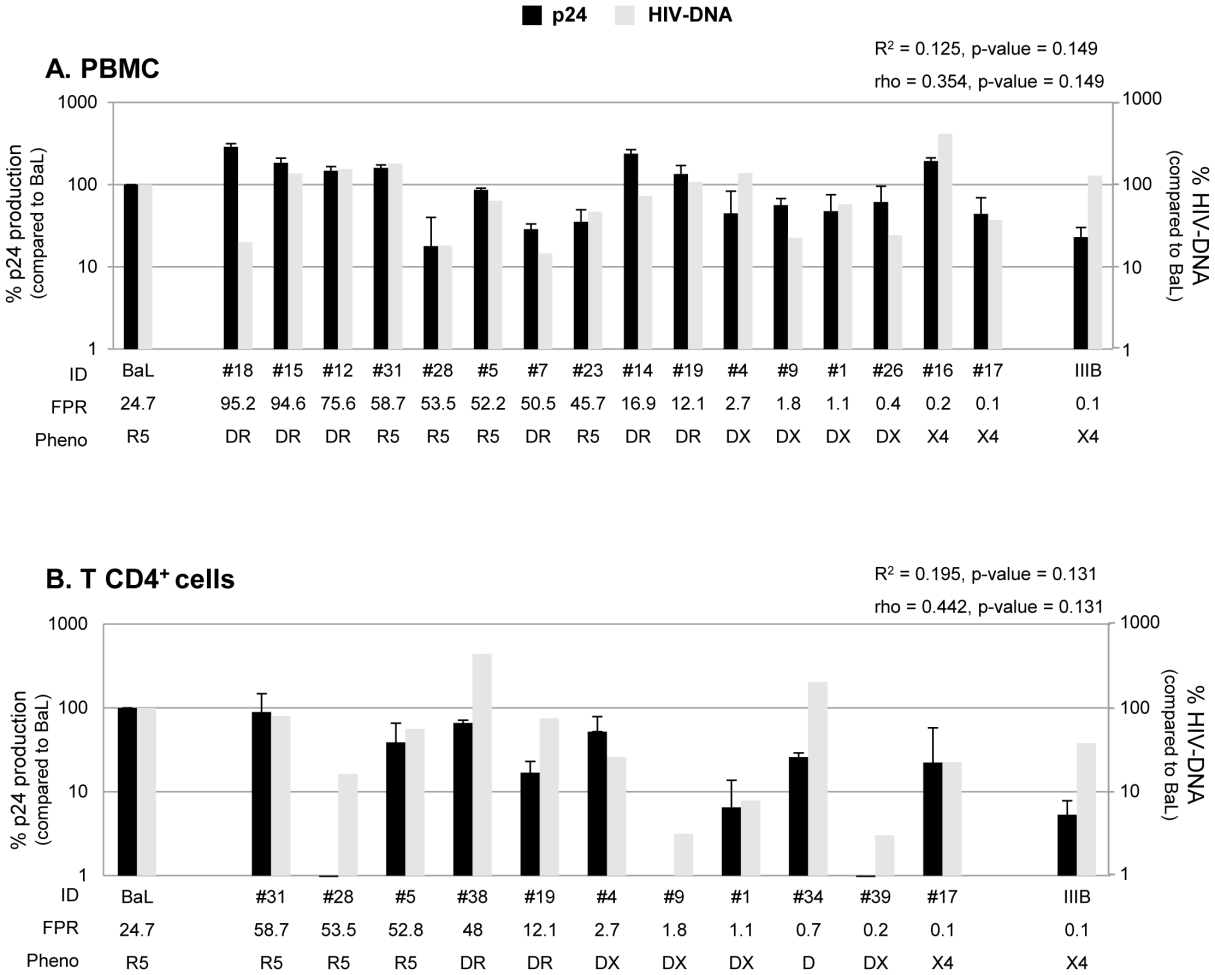
Comparison of HIV-DNA and viral replication in PBMC and CD4+ T-cells. The bars represents the percentage of HIV-1 DNA copies and p24 values (normalized per 10^6^ cells) in human primary cell types. Panel A and B: PBMC and CD4+ T-cells infected with 16,000 pg per 10^6^ cells after 7 days post infection, respectively. The percentage of p24 production and DNA copies of each isolate is calculated considering the replication of the control virus HIV_BaL_ as 100 (in PBMC: median p24 production of 45.506 pg/10^6^ and 555.000 HIV DNA copies/10^6^; in CD4+ T-cells: median p24 production of 357.561 pg/10^6^ and 2.5×10^6^ HIV DNA copies/10^6^ cells). The experiments for PBMC and CD4+ T-cells were done using cells from the same donor. Mean p24 production values of 3 biological replicates for each isolate are shown. Error bars indicating intra-experimental standard deviations are shown. Linear regression model and Pearson correlation coefficient were used to determine the strength of correlation between p24 production and HIV-DNA copies. R^2^ = Coefficient of determination; rho = Pearson correlation coefficient; p, two-tailed p value. The False Positive Rate (FPR) values are based on the V3 sequences and calculated with Geno2Pheno algorithm. The phenotypic tropism was evaluated on astroglioma U87MG-CD4^+^-CCR5^+^−/CXCR4^+^-expressing cells (DR: R5^+^X4; D: R5/X4; DX: R5/X4^+^).

In order to distinguish HIV infection and viral replication, HIV-DNA of infected cells was also quantified and compared to the p24 production. In PBMC cultures, no main differences were found at 7 days post infection by comparing the total HIV DNA copies with the p24 production (both normalized for 10^6^ cells) (Figure 5, panel A). Statistical analysis showed a non significant correlation between viral replication and HIV DNA values (R^2^ = 0.125, p-value = 0.149; rho = 0.354, p-value = 0.149).

**Figure 6:**
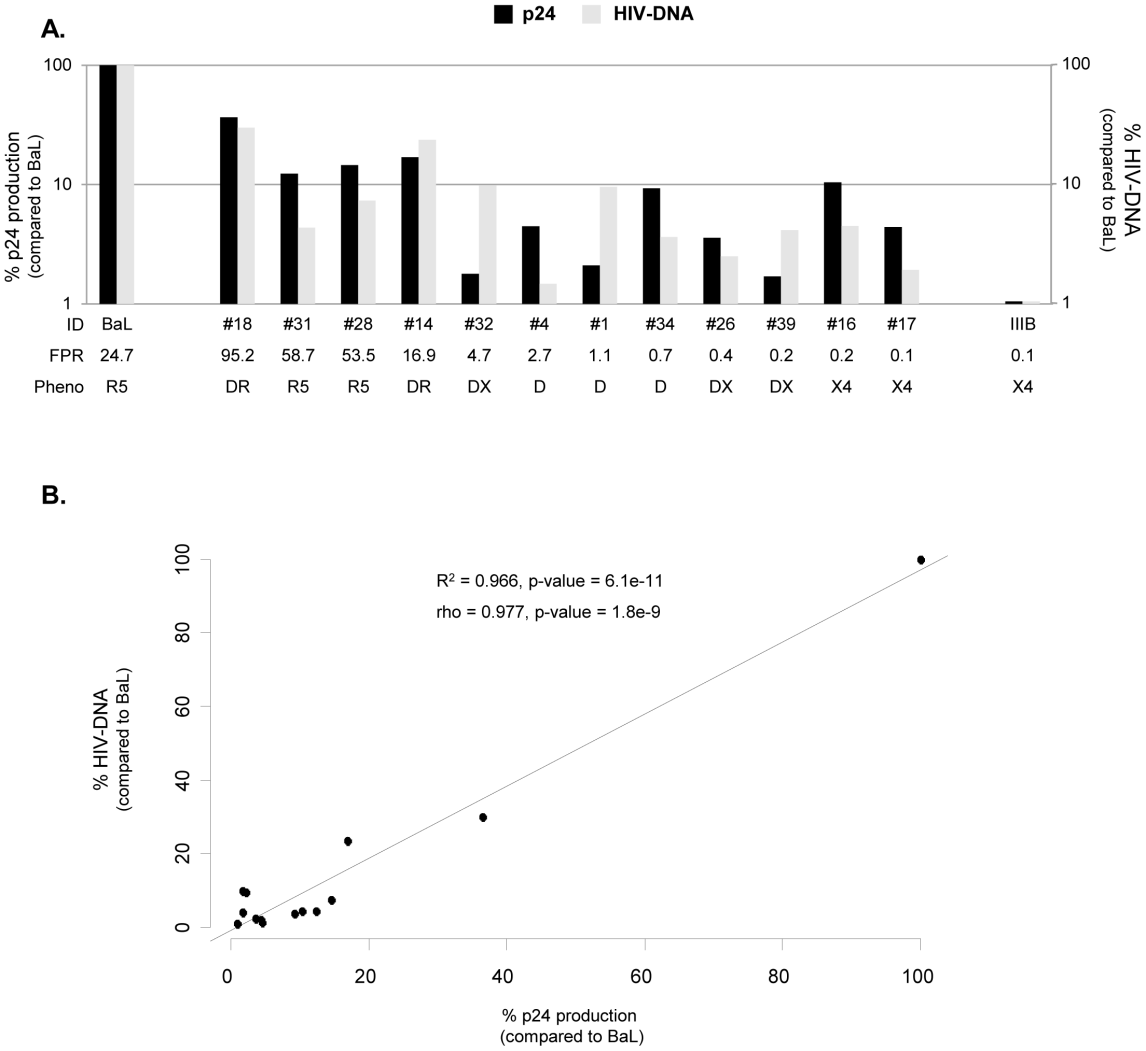
Comparison of HIV-DNA and viral replication in Macrophages. Panel A: The bars represents the percentage of HIV-1 DNA copies and p24 values (normalized per 10^6^ cells) in human primary MDM infected with 8,000 pg per 10^5^ cells after 14 days post infection. The percentage of p24 production and DNA copies of each isolate is calculated considering the replication of the control virus HIV_BaL_ as 100 (median p24 production of 499.310 pg/10^6^ and 9.3×10^6^ HIV DNA copies/10^6^). Panel B. The scatterplot shows the correlation between the replication capacity (p24 production) and HIV-DNA content, by using a simple linear regression model. Pearson correlation coefficient was also used to determine the strength of correlation between p24 production and HIV-DNA copies. R^2^ = Coefficient of determination; rho = Pearson correlation coefficient; p, two-tailed p value. The False Positive Rate (FPR) values are based on the V3 sequences and calculated with Geno2Pheno algorithm. The phenotypic tropism was evaluated on astroglioma U87MG-CD4^+^-CCR5^+^−/CXCR4^+^-expressing cells (DR: R5^+^X4; D: R5/X4; DX: R5/X4^+^).

The majority of isolates that replicated well also contained large amount of total HIV DNA. Isolates that replicated less efficiently were associated with a reduced amount of HIV DNA. Of interest, this rule does not apply for 3 isolates (#28, #9 and #39) that, in T-CD4^+^ cultures, poorly replicated (p24 production at 7 days post infection <12.5 pg/ml), but reached high levels of HIV DNA copies (406,714 copies/10^6^ cells; 78,869 copies/10^6^ cells; 76,049 copies/10^6^ cells, respectively (Figure 5, panel B). This suggests that these isolates were able to enter within CD4^+^ T cells and were more inclined to produce latent infection than other isolates (where the levels of DNA and p24 were more consistent). Overall, the statistical analysis showed a non significant correlation between viral replication and HIV DNA in CD4+ T cells (R^2^ = 0.195, p-value = 0.131; rho = 0.442, p-value = 0.131). Interestingly, in MDM, no significant differences were found by comparing total HIV DNA copies with the p24 production (both normalized for 10^6^ cells) (Figure 6, panel A), while there was a strong and significant direct correlation between viral replication and HIV DNA (R^2^ = 0.966, p-value = 6.1e-11; rho = 0.977, p-value = 1.8e-9) (Figure 6, panel B).

**Figure 7:**
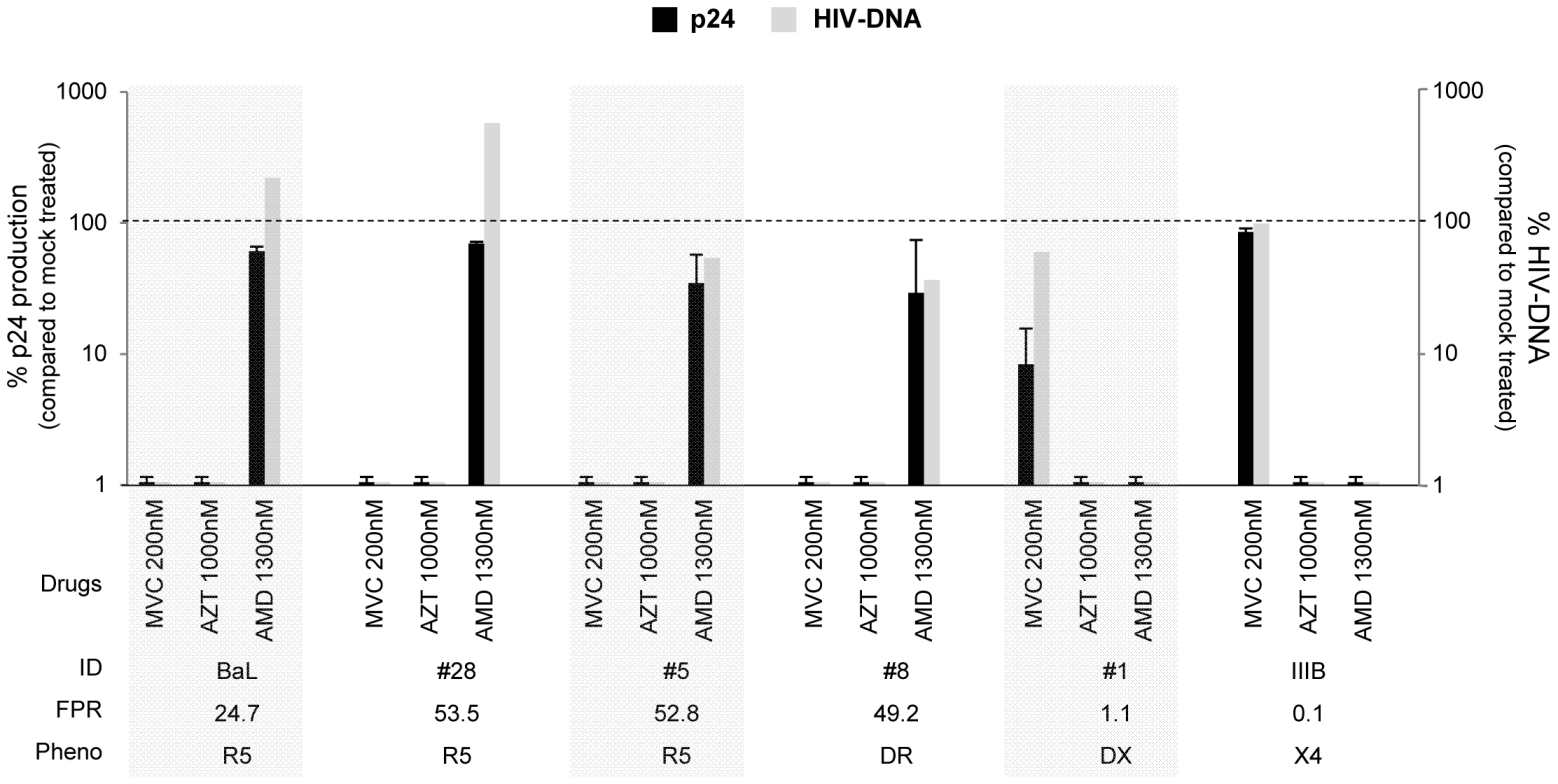
Inhibition of HIV DNA in presence of maraviroc in PBMC cultures. The bars represents the percentage of HIV-1 DNA copies and p24 values (normalized per 10^6^ cells) in the presence of AMD, AZT and maraviroc at different concentrations considering the untreated as 100. HIV_BaL_ is used as control virus. The p24 production is obtained from PBMC-cultures infected with 16,000 pg per 10^6^ cells after 7 days post infection. Data represent one of three experiments yielding similar results. Mean values of 3 biological replicates (p24 production) for each isolate are shown. Error bars indicating intra-experimental standard deviations are shown. The False Positive Rate (FPR) values are based on the V3 sequences and calculated with Geno2Pheno algorithm. The phenotypic tropism was evaluated on astroglioma U87MG-CD4^+^-CCR5^+^−/CXCR4^+^-expressing cells (DR: R5^+^X4; D: R5/X4; DX: R5/X4^+^).

As a confirmation that maraviroc inhibits both viral entry and therefore replication, in PBMC we observed that R5-isolates showed a median inhibition by maraviroc 200 nM of 99.8% for HIV DNA copies and 100% for p24 production (Figure 7). Similarly, in MDM, maraviroc 200 nM was able to completely inhibit HIV DNA copies (>99%) and p24 production (100%) in R5-isolates (data not shown).

According to p24 release, in PBMC maraviroc 200 nM was not able to well inhibit viral entry, in dual R5/X4^+^ or HIV-1_IIIB_ X4 tropic strains with FPR <5% (Figure 7).

Taken together, by comparing the total HIV DNA copies with the p24 production, MDM showed a strong and significant direct correlation between viral-replication and HIV-DNA, while in PBMC and CD4^+^ T-cells the direct correlation observed was not significant. Interestingly, some isolates not replicating in CD4^+^ T cells, where high levels of HIV DNA were observed, confirming the possibility of these cells to establish a latency phase after HIV-1 entry.

## Discussion

This work shows that HIV-1 clinical isolates exhibit a wide range of viral tropism and different rates of replication, and highlights the ability of maraviroc to inhibit not only pure-R5 but also dual/mixed-tropic viruses in human primary cells. These results underline the complexity and the heterogeneity of the viral population circulating in HIV-1 infected individuals, suggesting that using clinical isolates is a good model to appreciate the real viral contribution in mechanisms underlying HIV-1 pathogenesis *in vivo* (not fully appreciable when recombinant viruses are used).

We found that the majority of HIV-1 isolates efficiently replicated in PBMC and T cells. A weak correlation was observed between the efficiency of replication and viral tropism in both these cell types. In contrast, a stronger and significant correlation was observed in MDM. Consistent with higher expression of CCR5 co-receptor on their membrane, macrophages mainly sustained the replication of HIV-1 isolates with pure R5 or R5^+^/X4 tropism. On the other hand, the replication of all dual/mixed-tropic viruses with a genotypic FPR <20% was very poor in these cells.

Notably, in our panel of HIV-1 clinical isolates, viruses showing R5^+^/X4 tropism were found with higher prevalence (10/23). R5^+^/X4 isolates tended to replicate with comparable or even higher efficiency than isolates with pure R5 tropism. This phenomenon was more evident in macrophages, thus suggesting that dual/mixed-tropic isolates with FPR>20% can be characterized by a selective advantage compared to all other strains [64]. The efficient replication capacity of R5^+^/X4 isolates can have important pathogenic implications. Dual/mixed- tropic viruses emerge at later stages of infection in a significant proportion of individuals, differently from R5 viruses that predominate at earlier, asymptomatic stages of HIV-1 infection. Some studies have correlated the HIV-1 ability to replicate in macrophages with a faster disease progression [17], [65]–[67]. For instance, isolates from individuals with advanced HIV-1 infection or AIDS display enhanced replication capacity in macrophages compared to the isolates circulating during the early infection [65], [67].

We found that, in all primary cells, maraviroc inhibits viral replication of isolates not only with pure R5 tropism but also with dual/mixed tropism (mainly R5^+^/X4 and also to a lesser extent R5/X4 and R5/X4^+^) suggesting that CCR5-inhibitors are highly effective against all viruses with these dual-characteristics. This observation can be explained by a recent study showing a functional interaction of CCR5 and CXCR4 co-receptors in lymphocytes and monocytes, and the ability of CCR5 and CXCR4 antagonists to cross-bind both co-receptors [68].

Thus, the concept of HIV entry through one of the two coreceptors “separately” needs to be revised. Indeed, our *in vitro* results are also consistent with others obtained *in vivo:* a) from the A4001029-study, showing that maraviroc is effective also in some patients (27%) carrying dual-tropic viruses [69], b) from a recent case-report that demonstrates the ability of maraviroc to inhibit dual-R5 viruses in a dual/mixed HIV-1-infected patient [39].

The efficiency of maraviroc to inhibit HIV-1 replication in macrophages can have important pathogenic and clinical implications, in particular by reducing the dissemination of HIV in different body compartments and cellular reservoirs. This is particularly relevant in the central nervous system where maraviroc is known to efficiently penetrate [70]. In addition, macrophages at vaginal level have been shown to be permissive to HIV infection after the virus has translocated across the epithelium [71]. The high activity of maraviroc against macrophages might also contribute in controlling mucosal HIV-1 infection in sexual-transmission.

By analysing *in vitro* activity of CXCR4-antagonist AMD3100, we found the ability of this drug to inhibit the replication of pure-X4 and also some dual/mixed-tropic viruses (in particular R5/X4^+^ and R5/X4) in both PBMC and MDM. Again this is in line with another *in vivo* study, showing the ability of AMD3100 to suppress both X4-tropic and certain dual-tropic variants, that use more efficiently the CXCR4 coreceptor than the CCR5 [72].

Overall, the genotypic tropism testing on population-based V3 sequencing, is a valid tool for tropism determination in clinical practice (as also reported by the European Guidelines 2011 [73]). Indeed, all R5 genotypically predicted viruses were responsive to maraviroc *in vitro* in primary cells. This methodology has low cost and short turnaround, and has been recently shown to adequately predict virological response to maraviroc in drug-naïve patients of the MERIT Trial [74].

Finally, to better discriminate the viral entry and viral replication, we found no main differences in PBMC and CD4^+^ T cells by comparing the total HIV DNA copies with the p24 production in presence or absence of maraviroc. Some HIV-1 isolates not replicating in CD4^+^ T cells, reached high levels of HIV DNA, suggesting a latency phase after HIV-1 entry. The establishment of a latent infection has been generally considered to be dependent on the cell type infected by HIV [75]. Our finding confirms the existence of potential viral mechanisms regulating the entry into a latent phase in lymphocytes. This point deserves further investigation.

In conclusion, this study underlies the existence of complexity and heterogeneity among the HIV-1 population in terms of viral tropism. Our results reinforce the concept that HIV-1 tropism is a phenomenon not only dependent on the coreceptor usage but also on the replication capacity in different cell types. The majority of isolates efficiently replicated in both PBMC and CD4+ T-cells, regardless of their tropism, while macrophages mainly sustained the replication of HIV-1 isolates with pure R5 or R5^+^/X4 tropism. Moreover, the CCR5-antagonist maraviroc was active in both PBMC and macrophages against phenotypically pure R5 or R5^+^/X4 viruses, all R5 predicted by genotypic test. This supports the concept of extending the use of CCR5-antagonists to a spectrum of patients potentially larger than only those infected with a pure- R5 virus.

## Supporting Information

Table S1 Nucleotidic V3 sequences of isolates.
(DOCX)
