# Supplementary material for: Inhibition of Dual/Mixed Tropic HIV-1 Isolates by CCR5-Inhibitors in Primary Lymphocytes and Macrophages
Source: PLoS One. 2013 Jul 9;8(7):e68076. doi: 10.1371/journal.pone.0068076 (PMC3706609; doi:10.1371/journal.pone.0068076)
Supplement: Table S1 — Nucleotidic V3 sequences of isolates. (DOCX) [file pone.0068076.s001.docx]

| **Isolate ID** | **V3 Sequences** |
| --- | --- |
| #1 | TGTATAAGACCCAACAACAATACAAGAAGAAGGATAACTATGGGACCAGGGAGAGTATTATATACCACAGGAGAAATAATAGGAGATATAAGAAAAGCATATTGT |
| #4 | TGTACAAGRCCCAACAACAATACAAGAARAMGTATAWMTATGGGACCAKKGARAGCATWYKWTRCAACAGGASAMATAATAGGARATATAAGACAAGCAYATTGT |
| #5 | TGTACAAGACCCAACAACAATACAAGAAAAAGTATAMMTATAGGACCAGGGAGAGCATTTTATGCAACAGGAGAAATAATAGGAGATATAAGACAAGCACATTGT |
| #7 | TGTACAAGACCCAACAACAATACAAGAAAAAGTATAMMTATGGGACCAGGGAGAGCATTYTATGCAACAGGAGASRTAATAGGAGATATAAGACAAGCACATTGT |
| #8 | TGTACAAGACCTAAYAACAATACAAGAAAARGTATACATTTTGGACCAGGGCAAGCATGGTATACAACAGGACAARTAATAGGAGATATAAGACAAGCATATTGT |
| #9 | TGTACAAGACCCAACAACAATACAAGAAGAAGTATATCTATAGGACCAGGCAGAGCATTT---ACAACAAGACAAATAATAGGAGATATAAGAAAAGCATATTGT |
| #12 | TGTACAAGACCCARCAACAAYACAAGAAAAAGTATACCTATAGGRCCAGGGAGAGCATTTTATGCAACAGGAGACATAATAGGAGATATAAGAAAAGCACATTGT |
| #14 | TGCACAAGACCCAACAACAATACAAGAAAAAGTATACATATAGGACCAGGCAGAGCATTTCATGCAACAGGAGCAATAATAGGAGATATAAGACAAGCACATTGT |
| #15 | TGTACGAGACCCAACAACAATACAAGAAGAAGTATACCTATGGGACCARGCARGGCATTTTATGCAACAGGAGATATAATAGGAAATATAAGACAAGCACATTGT |
| #16 | TGTACAAGACCCAACMACYATACMGGAAGAAGAATAAGTATAGGACCAGGAAGAGCATTTCGTACA---GGTARAATAMTAGGAGACATAAGACAAGCACATTGT |
| #17 | TGTACAAGACCCAACACCAAGACAAGAAAAAGGATACATATAGGACCAGGCAGAGCATTTTATACAACAA---AAACAGTRAGAGATATAAGACAAGCACACTGT |
| #18 | TGTACAAGACCTAACAACAATACAAGAARAAGTATACATATAGGACCAGGYCAAGCATTTTATGCAACAGGAGACATAATAGGAGATATAAGACAAGCACAYTGT |
| #19 | TGTACAAGACCCAACAACAATACAAGAAAGGGTATACATATGGGACCAGGGAAAGCATTTTATGCAACAACAGATATAATAGGAGACATAAGAAAAGCATATTGT |
| #20 | TGTACAAGACCCAGCAACAAYACAAGCAAAAGTATACMKATAGGACCAGGCAGAGCATTTTATGCAACAGGAAGAATAATAGGAGATATAAGACAAGCACATTGT |
| #23 | TGTACAAGGCCCAACAACAACACAAGAAAAAGTATACATATAGSACCAGGGAGAGCATTTTATGCAACAGGASAAATAATAGGAGATATAAGAMAAGCACATTGT |
| #26 | TGTACAAGACCCGGCAACAATACAAGTAAAAGCATACGTATAGGAGCGAGGAAA---TTTTATGCAAGAGAAAGAATAATAGGAGACACAAGACGAGCACATTGT |
| #28 | TGTTCAAGACCCAACAACAATACAAGAAAAAGTATACATATAGGACCAGGCAAAGCATTTTATGCAACAGGAGAAATAATAGGAGATATAAGACAAGCATATTGT |
| #29 | TGTAYAAGACCCAACAATAATACAAGAAGAAGTGTGAGGRTMRGWYSARGAMAAACATTCTATGCAACAGGAGAYATAATAGGARACATAAGACARGCATATTGT |
| #31 | TGTACAAGACCTAACAACAATACAAGAAAAAGTATTAATMTAGGACCAGGGAGAGCATTTTATGCAACAGGAGATATAATAGGAGATATAAGAMARGCACATTGT |
| #32 | TGTATGAGACCCAACAACAATACAAGAAAAAGTATACATATGGGACCAGGCAGAGCATTTTATACAACAGGAGATATAATAGGAGATATAAGACAAGCACATTGT |
| #34 | TGTATRAGGCCMAACAAYAATACAAGAAAAGGTATACGTATAGGACCAGGGAGAGCAGTCTATGCAGCAGAGAAAATAATAGGAGATATAAGACAAGCACAYTGT |
| #38 | TGTACAAGACCCAGCAACAATACAAGAAGAGGTATACATATAGGACCAGGGAGAGCATTTTATGCAACAGGAGCCATAACAGGAGATATAAGACAAGCACATTGT |
| #39 | TGTACAAGACCCAACGCAATTACAAGAGGAAGGATACATATAGGACCAGGGAGAGCATTTCATGCAACAA---AAACCATAAAAGATATAAGACAAGCATATTGT |
